# Evaluation of Changes in Cariogenic Bacteria in a Young Moroccan Population with Fixed Orthodontic Appliances

**DOI:** 10.1155/2018/5939015

**Published:** 2018-11-27

**Authors:** A. Marda, S. Elhamzaoui, A. El Mansari, K. Souly, F. Farissi, M. Zouhdi, F. Zaoui, L. Bahije

**Affiliations:** ^1^Research Team in Microbiology, Faculty of Medicine and Pharmacy, Mohamed V University, BP 6203, Rabat, Morocco; ^2^Department of Microbiology Faculty of Medicine and Pharmacy, Mohamed V University, BP 6203, Rabat, Morocco; ^3^Faculty of Dental Medicine Graduate, Mohammed V University, Rabat, Morocco; ^4^Laboratory of Microbiology, Ibn Sina University Medical Center of Rabat, Rabat 10100, Morocco; ^5^Research Team in Molecular Virology and Oncobiology, Faculty of Medicine and Pharmacy, Mohammed V University, BP 6203, Rabat, Morocco; ^6^Department of Research in Biomaterials and Saliva Biomarkers, Department of Dentofacial Orthopedics, Faculty of Dental Medicine, Rabat-Institute, Hospital Center of Ibn-Sina, Mohammed V University (UM5) of Rabat, BP 6212, Madinat Al Irfane, Rabat, Morocco

## Abstract

Fixed orthodontic appliances hinder the maintenance of proper oral hygiene and result in dental plaque accumulation. Many studies report that qualitative changes in the dental flora occur after initiating the orthodontic treatment, but there is a paucity of literature on the same topic among Moroccan orthodontic patients. The aim of this study was to evaluate the changes of the oral microbial flora during the orthodontic treatment period of a young Moroccan population. *Materials and Methods*. Dental plaque samples of 18 patients, who were randomly selected before the placement of orthodontic appliances, were collected to isolate and identify the bacterial species involved using classical bacteriological methods for species' culture and identification. The reading was recorded at T0 before placement of the device. New samples were taken again one month later and then three months afterwards, where the readings were recorded as T1 and T2, respectively. The culture was made via Columbia Agar with 5% sheep blood, Todd Hewitt Broth, and Schaedler medium containing vitamin K3. Bacterial species were identified using API-20 Strep for *Streptococci* and API-20 A for anaerobic bacteria. The phoenix system was used for identification. *Results*. After three months of orthodontic treatment, the increase in the frequency of *Streptococcus sobrinus* and *Streptococcus mitis* were significant (0.01 and 0.02, respectively) as well as for *Lactobacillus* (0.03). No significant difference was recorded for other bacterial species. *Conclusion*. There is a significant qualitative change in oral microorganisms after three months of orthodontic treatment, especially for bacteria that are incriminated in caries formation.

## 1. Introduction

Fixed orthodontic appliances are considered to jeopardize dental health due to the accumulation of oral microorganisms that may cause enamel demineralization, which is clinically visible in the form of white spot lesions [1]. Biofilm formation on fixed orthodontic appliances occurs due to complex interactions between yeast, bacteria, nutrients, and saliva or even serum proteins [2].

Oral *Streptococcus* is isolated in 50–80% of orthodontic patients as a common cause of decalcification due to the accumulation of cariogenic plaque around the brackets and its progression into carious lesions in such patients [3].

Numerous studies have investigated the influence of orthodontic therapy and appliances on the oral microbial flora. The original aim of the present study was to assess the qualitative changes of cariogenic bacteria in a representative sample of Moroccan young adults.

The placement of fixed orthodontic appliances on teeth results in iatrogenic side effects [2]. During the treatment, dental lesions become difficult to access, resting pH decreases, the volume of dental plaque is higher, and bacterial flora undergoes qualitative change. All of these factors increase caries risk. Several investigations have shown that changes in the dental flora appear after the initiation of orthodontic treatment [3], such as a higher prevalence of oral streptococci especially *Streptococcus mutans* and *Strepcoccus mitis*, which are part of the normal bacterial flora of the oral cavity.

The flora only becomes pathogenic when it is put under circumstances that lead to a frequent and continuous acidification of dental plaque [1] and higher counts of *Lactobacillus* species, which are closely associated with dental caries. However, there is a scarcity of literature on this topic among Moroccan orthodontic patients.

The aim of the current study was to compare the oral microbial flora among Moroccan patients treated with orthodontic appliances by evaluating the status of this bacterial environment before and after bracket placement.

## 2. Materials and Methods

### 2.1. Subjects

After a complete oral examination, thorough oral hygiene instructions, and plaque control measures, a total number of eighteen young patients scheduled for orthodontic treatment, age group 16–26, were selected randomly from the Department of Orthodontics, Center of Consultations and Dental Treatments, Rabat, Morocco, during the period from October 2015 to June 2016.

Sampling was carried out via a questionnaire while taking into account the following inclusion criteria: patients with permanent dentition, no clinical signs of periodontitis, no history of systemic diseases, and no administration of antibiotics at least 3 months prior to the treatment. Exclusion criteria included smoking, pregnancy, poor general health, history of periodontal therapy, and antibiotic use.

Dental plaque samples were collected before placement of orthodontic appliances to determine the oral carriage of *Streptococci* and *Lactobacillus* in these patients, and the first reading was recorded as T0. After that, the placement of titanium nickel orthodontic wires (G&H Wire Co., 0.018″ (0.45 mm) NiTi wire comprising 55% nickel and 45% titanium) and brackets (Ormco Corporation, Glendora, CA, USA) was done by only one operator to avoid interoperator bias. After a period of one month and then three months later, the plaque samples were collected again from the same site, and the second and third readings were recorded as T1 and T2, respectively. The mentioned intervals were chosen based on the assumption that a sufficient bacterial colonization would take place after the placement of orthodontic appliances [1]. All patients had been taught and demonstrated the modified Bass brushing technique using a manual toothbrush (Oral B, Procter & Gamble, Cincinnati, OH, USA) and were motivated to meticulously follow throughout the treatment period [4].

### 2.2. Salivary Microbial Procedures

Dental plaque was collected according to the protocol from the dental surface with a sterile curette followed by rapid transfer of the samples to Amies transport media MAST (DM030) and to anaerobic bacteria culture media (Schaedler broth with vitamin K) [5].

The recipients were immediately sent to the microbiology laboratory where they were centrifuged for 30 seconds and diluted at 1/100 in physiological serum. 50 *µ*l aliquots were spread on Mitis Salivarius Agar (HiMedia des Laboratoires Pvt. Ltd., Mumbai, India), Columbia agar with 5% sheep blood, and Schaedler medium containing vitamin K3 (Schaedler K3 medium, broth, and agar). After 24–48 hours of incubation at 37°C for aerobic bacteria and 2–7 days for anaerobic bacteria, the developed colonies were morphologically examined. Each type of colony was subcultured on nonselective growth media to obtain pure cultures. Isolated bacterial colonies were identified based on their morphotinctorial characteristics (Gram-stained smears) and growth characteristics on media, and they also underwent biochemical tests. The species were identified using Rapid ID32 STREP galleries biochemical and enzymatic tests (BioMerieux, SA) for *Streptococci* and API-20A (BioMerieux, SA) for anaerobic bacteria. To ensure the bacterial identification, we used the automaton Phoenix (Becton Dickinson Phoenix 100 Microbiology Automaton© Sogemed).

The bacterial strains were preserved in cryotubes by freezing. In each 1.5 ml cryotube, 500 *μ*l of bacteria and 500 *μ*l of bacterial stock glycerol were mixed together and stored in the freezer (20 to 80°C) to use in case further research via molecular diagnostic-genotypic methods was conducted in the future.

### 2.3. Statistical Analysis

The statistical analysis was performed using the SPSS 13.0 software package. The data were analyzed using descriptive statistics. Fisher's exact test for qualitative variables and the Friedman test for quantitative variables were used to determine the significance of differences between the three groups. The level of statistical significance was set at *P* < 0.05.

## 3. Result

### 3.1. Descriptive Statistics of the Study Population

The study included 18 patients, 11 women (61%) and 7 men (39%). The age was between 16 and 26 years. The average age of all patients was 21.83 ± 3.48 years. There was no significant difference between men and women.

### 3.2. Macroscopic and Microscopic Appearance

**Figure 1:**
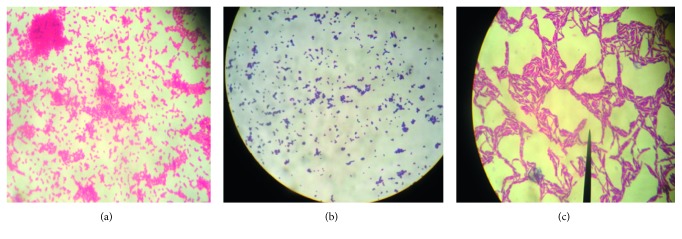
(a) Gram-negative *Bacillus* (optical microscope obj. ×100). (b) Gram-positive cocci (optical microscope obj. ×100). (c) Gram-positive *Bacillus* (optical microscope obj. ×100).

The observation of the samples with the naked eye and the optical microscope shows that they are neither hematological nor purulent. After the isolation of the colonies, Gram-positive cocci, Gram-negative bacilli, and Gram-positive bacilli were obtained (Figure 1).

Table 1 presents the frequencies of analyzed bacteria in samples collected from the sample group in T0, T1, and T2.

No significant difference in frequencies was registered for most of the analyzed bacteria, from T0 to T1 and T1 to T2. However, the frequencies *of Streptococcus sobrinus* and *Streptococcus mitis* have increased significantly, from T0 to T2 (*P*=0.016, *P*=0.021).

As to *Lactobacillus*, the frequency has increased significantly from T0 to T2 (*P*=0.031) and insignificantly from T0 to T1 and T1 to T2. As for enterobacteria, we notice that there is an appearance after 3 months of installation of the fixed orthodontic apparatus of *Enterobacter cloacae* and *Klebsiella pneumoniae* (Table 1).

## 4. Discussion

In this study, we found that the frequencies of isolation of bacteria in the oral cavity in patients before orthodontic appliance placement were greater than those measured after 1 month and 3 months of orthodontic appliance placement, respectively. The placement of orthodontic appliances makes it difficult to remove dental plaque by the simple use of a toothbrush and dental floss.

In T0 and T2, we noticed an increase in cariogenic oral microorganisms, especially *Streptococcus mitis*, *Streptococcus sobrinus*, and *Lactobacillu*s. This finding was statistically significant (*P*=0.021, 0.016, 0.031).

According to Chang et al., the increase in oral *Streptococcus* following placement of orthodontic appliances can be explained by the irregular nature of their surfaces, which promotes the growth of acidogenic bacteria that prefer to grow on hard surfaces [6]. Batoni et al. showed that orthodontic treatment caused a modification in the oral flora and was associated with elevated counts of cariogenic bacteria in both the dental plaque and saliva [2].

Our finding can be explained by the qualitative change in oral ecology. In other words, it is due to a change in the bacterial composition of dental plaque in patients wearing orthodontic appliances. This result is in line with findings of other studies (Kanaya in 2005 and in 2007 [5, 6]) which confirmed that orthodontic treatment creates a favorable environment for both quantitative (fast rise in the volume of plaque) and qualitative changes [7]. Kitada et al. in 2009 found in 42 patients, 1 case of *Klebsiella pneumoniae* and 6 cases of *Enterobacter cloacae*. In our study, we also distinguished the appearance of these germs after 3 months of fitting the orthodontic apparatus [8]. The literature results show that *Klebsiella pneumoniae* and *Enterobacter cloacae*, which are anaerobic and do not need air or dioxygen to function, are among the major causes of halitosis [9]. Rosembloom and Tinanoff found that orthodontic treatment would result in a change in oral flora with a remarkable increase in cariogenic bacteria counts (including oral streptococci) in dental plaque and saliva [10]. In our study, we have seen a proliferation of *Streptococcus mitis* and *Streptococcus sanguinis* which are cariogenic bacteria.

Some investigations have presented new data on the duration of microbial salivary changes due to the placement of fixed orthodontic appliances [11]. A significant increase in the cariogenic microorganisms *Streptococcus mutans* and *Lactobacillus* in saliva was found after starting fixed orthodontic therapy.

Indeed, these appliances serve as bacterial retention sites. Biofilm formation on the materials used in conservative dentistry and prosthetic dentistry has been investigated by several studies. However, very few studies have examined the interactions between the oral bacteria, particularly *Streptococcus mutans*, which are the main cariogenic bacteria, and the different materials used in orthodontics.

In vivo studies generally revealed an increase in dental plaque [12] with a specific rise in oral S*treptococcus* and *Lactobacillus* counts in plaque and saliva during the orthodontic treatment period [13]. According to these authors, orthodontic appliances are retention sites that promote colonization by certain cariogenic bacteria: oral streptococci and lactobacilli [14]. The characteristics of dental plaque retention on appliances are the cause of a severe development of caries in unusual parts of the teeth, like the vestibular surfaces [15].

## 5. Conclusion

Our study consisted of evaluating the quality of bacterial dental plaque before and after the placement of fixed orthodontic appliances on a population of young Moroccan adults. Fixed orthodontic appliances promote the colonization of oral bacteria such as the following cariogenic bacteria: *Streptococcus mitis*, *Streptococcus sobrinus*, and *Lactobacillus*, hence the need to regularly maintain good oral hygiene.

## Figures and Tables

**Table 1:** Frequency of analyzed bacteria in dental plaque during three phases: before the placement of fixed appliances (T0), one month after the placement of fixed appliances (T1), and three months after the placement of fixed appliances (T2).

Bacteria	T0	T1	T2	*P*-value		
	*N* (%)	*N* (%)	*N* (%)	T0 vs T1	T1 vs T2	T0 vs T2
*Streptococcus sobrinus*	7 (38.9)	9 (50)	14 (77.8)	0.5	0.125	0.016^*∗*^
*Streptococcus oralis*	2 (11.1)	2 (11.1)	3 (16.7)	1	1	1
*Streptococcus sanguinis*	2 (11.1)	3 (16.7)	6 (33.3)	1	0.375	0.219
*Streptococcus mitis*	2 (11.1)	3 (16.7)	10 (55.6)	1	0.065	0.021^*∗*^
*Streptococcus salivarius*	3 (16.7)	3 (16.7)	3 (16.7)	1	1	1
*Lactobacillus*	2 (11.1)	6 (33.3)	8 (44.4)	1	0.63	0.031^*∗*^
*Klebsiella pneumoniae*	0	0	2 (11.1)	—	—	—
*Enterobacter cloacae*	0	0	3 (16.7)	—	—	—

^*∗*^ *P* < 0.05 compared to T0.

## Data Availability

The data used to support the findings of this study are available from the corresponding author upon request.
